# Urban Life Shapes Genetic Diversity in the Green Anole, *Anolis carolinensis*

**DOI:** 10.1111/mec.70057

**Published:** 2025-07-29

**Authors:** Yann Bourgeois, Simon Lailvaux, Stéphane Boissinot

**Affiliations:** ^1^ UMR DIADE, University of Montpellier, CIRAD, IRD Montpellier Cedex 5 France; ^2^ Department of Biological Sciences University of New Orleans New Orleans Louisiana USA; ^3^ Division of Science New York University Abu Dhabi Abu Dhabi UAE; ^4^ Center for Genomics and Systems Biology New York University Abu Dhabi Abu Dhabi UAE

**Keywords:** adaptation, anthropization, conservation genetics, *HOX* genes, molecular evolution, population genetics—empirical

## Abstract

Urbanisation presents unique environmental pressures that drive rapid evolutionary adaptations, particularly in species inhabiting fragmented and anthropogenised landscapes. In this study, we investigate the genomic differentiation between urban and non‐urban populations of *Anolis carolinensis* , focusing on two main aspects: (1) the effect of habitat fragmentation on inbreeding and mutational load and (2) genomic adaptation to the urban habitat. We found that urban populations can exhibit a reduced mutational load, which is a direct consequence of systemic inbreeding. Using genome‐wide scans of selection and analyses of genetic diversity, we identify key genomic regions exhibiting significant divergence between urban and non‐urban populations. These regions are enriched for genes associated with immunity, behaviour and development, suggesting that urban adaptation is polygenic and involves traits related to stress response, locomotion and thermoregulation. Scans for association with the urban environment reveal a large genomic region in chromosome 2 encompassing the *HOXC* gene cluster. We also detect a signal of both association and increased differentiation on chromosome 1 in a region previously identified as a candidate for convergent adaptation in another *Anolis* species, *A. cristatellus* . Although evidence for convergent evolution at the gene level remains limited, potential signatures of urban adaptation in loci involved in immune response, locomotion and behaviour support the hypothesis that urban environments exert similar selective pressures across species. These results provide evidence for redundancy and polygenic adaptation and highlight the complexity of urban evolution. Future work with denser population sampling and time‐series data will be essential to confirm the role of urban selective pressures and track the genetic dynamics of urban populations over time.

## 1 Introduction

Urbanisation drastically alters environments (Seto et al. 2010), introducing novel biotic and abiotic conditions that can profoundly shape the evolutionary trajectories of species (Hendry and Kinnison 1999). Cities present unique ecological challenges, including habitat fragmentation, pollution, altered thermal landscapes, and novel predator–prey dynamics (Donihue and Lambert 2015). These pressures often lead to rapid adaptation, with some organisms thriving in urban environments, whereas others decline (McKinney 2008). There has been a growing interest in the study of urban environments because urban areas, as replicated “natural experiments,” provide an opportunity to study evolutionary changes over short timescales and test for convergent evolution (Miles et al. 2021; Winchell et al. 2023).

Recent studies have documented that urban habitats are associated with genetic and phenotypic adaptations, such as modifications in immune function, locomotion and thermoregulation, highlighting the importance of selective pressures unique to urban environments (Frantz et al. 2012; Halfwerk et al. 2018; Lambert et al. 2013; Rees et al. 2009; Slabbekoorn and Peet 2003; Winchell et al. 2023). However, the genetic bases of such adaptations remain elusive and poorly quantified in many species (Charmantier et al. 2024). This can be explained by the relatively recent application of population genomic tools to the study of urban adaptation. Genome‐wide scans of selection and association can be used in a “bottom‐up” manner (Ross‐ibarra et al. 2007): by naively scanning the genome for signatures of selection, one may identify new genes of interest and connect them to relevant phenotypes. This approach presents several issues that are not exclusive to the study of urban habitats but are common to any study of the proximal causes of adaptation. Some of these issues include the confounding effects of background and linked selection, which may reduce diversity and increase divergence between urban and non‐urban populations in the absence of any local adaptation (Haasl and Payseur 2016; Jensen 2023; Jensen et al. 2019; Perrier and Charmantier 2019). Other issues include the poor knowledge of demographic history, the difficulty in identifying strong signatures of selection for complex and polygenic adaptation (Wellenreuther and Hansson 2016), the lack of functional validation and storytelling (Pavlidis et al. 2012; Perrier and Charmantier 2019). Although simulation tools have become more widely used and offer a way to test for the robustness of signals of selection (Harris et al. 2018), the recent and intense dynamics typically encountered in urban populations can make it difficult to properly account for all sources of biases. Storytelling and other confounding effects may be partly alleviated by testing hypotheses grounded in the functional knowledge gathered for well‐studied candidate genes (“top‐down” approach [Ross‐ibarra et al. 2007]).

Another aspect to consider when investigating urban populations is their size and fragmentation. Small, isolated populations, such as those in urban habitats, are at risk of increased inbreeding, which can result in inbreeding depression and the expression of recessive deleterious mutations in their homozygous form (Keller and Waller 2002; Laenen et al. 2018; Lynch et al. 1995). This can lead to a decrease in the population's overall fitness, further contributing to population decline. The success or extinction of urban populations may well depend on such processes. In species having faced recent, strong and repeated bottlenecks (such as the alpine ibex), purging of strongly deleterious variants has been observed (Grossen et al. 2020). Such a pattern may also be expected in established urban populations, although it has not been investigated in this context to the best of our knowledge.

Lizards of the genus *Anolis* have become a model for studying how organisms adapt to urban habitats. Most studies on the ecological and evolutionary consequences of urbanisation have focused on Caribbean anoles, either in their native range where they are exposed to extreme heat, or in an invasive context (e.g., Floridian populations of Caribbean anoles). These studies have identified a number of morphological and physiological changes associated with urban habitats (Campbell‐Staton et al. 2021; Howell et al. 2022; Prado‐Irwin et al. 2019; Thawley et al. 2019; Winchell et al. 2016, 2018) and in some cases have determined the underlying genomic changes responsible for urban adaptation (Campbell‐Staton et al. 2020). Interestingly, several studies have documented examples of genomic parallelism in urban populations (Campbell‐Staton et al. 2020; Winchell et al. 2023). In a landmark study, Winchell et al. found evidence of genomic parallelism among 3 pairs of urban/non‐urban populations of *Anolis cristatellus* . The signature of genomic parallelism was found at genes involved in immunity, wound healing, neural function, metabolism and skin development, that is, functions that are relevant to adaptation to an urban environment. It remains unclear, however, if genomic parallelism would be observed among divergent species or among ecomorphs. Testing whether these signatures are shared across species would reinforce the case for convergent evolution, and expose the link between genes and the phenotypes that have been extensively studied in anoles (Campbell‐Staton et al. 2020; Glor et al. 2005; Lailvaux et al. 2004; Lapiedra et al. 2018; Losos 2009; Tollis et al. 2018) particularly locomotion, development and behaviour.

The green anole, *Anolis carolinensis* , is the most well‐studied species in the genus *Anolis* and has been a model organism in ecology and evolution for over a century (Lovern et al. 2004). Today, green anoles also present an excellent, albeit under‐utilised model to study questions relating to urban evolution since it exhibits the largest distribution of any anole species and is commonly found in city parks and gardens across its range in the United States. It is also the only anole that has successfully colonised temperate habitats. Having diverged from *Anolis cristatellus* 35–40 MY ago, *A. carolinensis* and *A. cristatellus* differ in morphology, *A. cristatellus* being a typical trunk‐ground ecomorph and *A. carolinensis* a trunk‐crown ecomorph. Comparing the adaptation to urban habitat between these two species will thus allow us to determine if genomic parallelism can extend to species that diverged a long time ago, differ in morphology and are adapted to different climates (tropical for *A. cristatellus* and temperate for *A. carolinensis* ).

Urbanisation appears to impact the ecology of green anoles in a variety of ways, ranging from morphology and demography to display and escape behaviour; territoriality; and sexual selection (Weber et al. 2021; reviewed in [Lailvaux 2020]). Male–male competition in particular appears to be heightened in urban green anole populations, likely because of limited, concentrated resources (Bloch and Irschick 2006). This in turn places stronger emphasis on physical traits such as body size and biting ability for determining territorial success and mating opportunities (Husak, Lappin, and Van Den Bussche 2009; Lappin and Husak 2005; McMillan and Irschick 2010) emphasising how urban habitats can reshape natural behaviours. For example, previous studies have reported the existence of two life‐stage morphs among adult male green anoles in the New Orleans, Louisiana area (Husak et al. 2007; Husak, Irschick, et al. 2009; Lailvaux et al. 2004).

The present study builds on previous findings by examining the possible impact of urbanisation on genomic diversity in *A. carolinensis* in Washington Square Park, New Orleans (Weber et al. 2021). This population has been followed over several years (Weber et al. 2021) and provides an ideal framework for integrated studies. We use a large (87) cohort of urban anoles sequenced at low depth (3×) to explore two key questions: (1) How does urban life, characterised by fragmentation, influence population structure, inbreeding and mutation load? (2) Are there signs of recent, possibly parallel adaptation to the urban environment, particularly regarding immune, locomotion, or heat resistance genes identified in other species? The goal here is to determine whether adaptation in urban anoles involves diffuse polygenic responses and/or strong selection at a few loci of major effect.

## 2 Methods

### 2.1 Sample Collection and Sequencing

A total of 87 male anoles from Washington Square Park (N29.965005°, W90.057302°, New Orleans, Louisiana, USA) were selected for low‐depth sequencing (target 3×). These males were part of a larger sample of green anole lizards collected in the Spring and Fall of each year from 2010 to 2014 (see Lailvaux 2020 for a detailed description of the sample site and sampling regime). Sixteen more samples (males) were also collected from neighbouring localities in non‐urban areas surrounding New Orleans to provide a contrast for tests of selection (Table S1). We added eight more samples obtained from previous studies, two of them close to New Orleans, and six others from outside Louisiana. All samples belonged to the same genetic cluster (Gulf‐Atlantic) described in previous studies (Bourgeois et al. 2019; Bourgeois and Boissinot 2019; Campbell‐Staton et al. 2012; Manthey et al. 2016; Tollis and Boissinot 2014). Tissue samples consisted in tail tip of no more than 10 mm that were collected with sterilised scissors and placed it into a vial of 95% ethanol. DNA was isolated from ethanol preserved tissue using was extracted using the DNeasy blood and tissue kit (Qiagen, Valencia, CA) and purified with Ampure beads per the manufacturers' protocol. Illumina TRU‐Seq paired end libraries were generated using 200 ng of DNA per sample and paired‐end sequenced (2× 150 bp) at the NYUAD Sequencing Core (http://nyuad.nyu.edu/en/research/infrastructure‐and‐support/core‐technology‐platforms.html; Last accessed on August 12, 2024) with an Illumina HiSeq 2500. Read quality was assessed with FastQCv0.11.X (http://www.bioinformatics.babraham.ac.uk/projects/fastqc; Last accessed on August 12, 2024) and Trimmomatic (Bolger et al. 2014) was subsequently used to remove low quality bases, sequencing adapter contamination and systematic base calling errors. Specifically, the parameters “trimmomatic_adapter.fa: 2:30:10 TRAILING:3 LEADING:3 SLIDINGWINDOW:4:15 MINLEN:36” were used. Reads were aligned using BWA‐MEM v and PCR duplicates were removed using GATK/PICARD tool MarkDuplicates (https://broadinstitute.github.io/picard/; Last accessed on August 12, 2024).

### 2.2 Variant Calling and Annotation

Because the data we obtained are low‐depth (2× for urban anoles), we used the genotype likelihood framework embedded in the ANGSD v0.940 toolkit (Korneliussen et al. 2014) instead of using called genotypes. We use the SAMTools model of genotype likelihoods (option ‐GL 1). We always used filters on mapping quality and removed not properly paired reads with multiple hits and non‐primary alignments (‐uniqueOnly 1 ‐remove_bads 1 ‐only_proper_pairs 1 ‐C 50 ‐baq 1 ‐minMapQ 20). Bases with a Phred quality score lower than 30 were excluded from the inference (‐minQ 30). We also removed sites with a Phred genotyping quality score lower than 30 (‐SNP_pval 1e‐3, except when monomorphic sites were required for inference, see below), an average depth of coverage higher than the 99% quantile observed in each population (‐setMaxDepth) and a maximum proportion of missing data of 33%.

We also produced a VCF file on all 103 (87 + 16) Louisiana individuals using bcftools v1.16 (Danecek et al. 2021) to obtain a list of variants to annotate with SNPEff v5.2 (Cingolani et al. 2012). The latter programme produces an annotated VCF for all SNPs, classified into non‐coding and coding variants, the latter being classified as variants of low (synonymous variants), moderate (usually non‐synonymous variants), or high effects (typically premature stop codons).

### 2.3 Relatedness, Inbreeding and Structure

We used ngsRelate (Hanghøj et al. 2019) to estimate relatedness between samples using genotype likelihoods, using two measures of relatedness (scaling from 0 to 1.0, *R*_AB_) and kinship (scaling from 0 to 0.5, Θ) that have given robust results in a broad range of experimental settings and generally agree well with pedigrees (Hauser et al. 2022). We also examined the KING‐robust estimator of kinship (Waples et al. 2019), which does not rely on population allele frequencies but instead examines the two‐dimensional site frequency spectrum (2D‐SFS) of pairs of individuals to assess relatedness. We excluded seven samples with fewer than 50% of sites covered (Table S1, samples 44, 311, 316, 348, 352, 357 and 371).

We estimated the inbreeding coefficient *F* for all individuals using the method implemented in ngsF (Vieira et al. 2013). The method also takes genotype uncertainty into account to produce unbiased estimators of inbreeding. We ran the algorithm 20 times using the fast EM method and retained the estimates with the highest likelihood.

We defined a set of ‘unrelated’ samples by removing the individual with the lowest depth of coverage from each pair with a relatedness coefficient *R*_AB_ > 0.25 and kinship coefficient Θ > 0.125, corresponding to putative first and second‐degree relatives (see Section 3).

We ran PCAngsd (Meisner and Albrechtsen 2018) to describe population structure through a principal component analysis. We included in this analysis the set of ‘unrelated’ urban individuals and the 16 samples from outside the city and used markers with a minimal allele frequency of 0.05.

### 2.4 Mutation Load

To estimate mutation load (autosomal) in urban anoles, we first calculated Site Frequency Spectra (SFS) for three categories of variants (Low, Moderate and High Effect) classified by SNPEff, using the ‐Sites option in ANGSD. We used realSFS to obtain the spectra from the saf files generated in ANGSD with the option ‐doSaf 1. First, we estimated the SFS for the unrelated urban individuals and other Louisiana individuals separately. To reduce the raggedness of the spectrum estimated by realSFS, we projected the SFS down using ∂a∂i (Gutenkunst et al. 2009). We projected the frequency spectrum of the urban population down to 64 alleles per locus, and the non‐urban population to a count of 24 alleles. These numbers were chosen as they corresponded to two times the minimum number of individuals with no missing data in each population sample (given our filtering rule of no more than 33% missing data). We also estimated the joint (or 2‐dimensional, 2D) SFS between these two groups to compare the mutation load in the urban individuals compared to the rest of Louisiana. To do so, we calculated the *R*_*xy*_ statistics (Do et al. 2015), the ratio between the number of derived mutations in a genome from population X that are not seen in another genome from population Y, and the number of derived mutations from population Y not found in population X, averaged through all possible pairwise comparisons. This statistic is standardised ($R_{\mathrm{XY}}^{\prime }$) by the statistic observed at a set of putatively neutral markers to correct for past demography. On the basis of allele frequency spectra and values for the other statistics, we normalised using *R*_*XY*_ values obtained for 20,000 polymorphic intergenic variants. We considered the reference genome (obtained from an individual collected in South‐Carolina [Alföldi et al. 2011]) to be sufficiently distant to be used as an outgroup when polarising variants into ancestral (reference) and derived. Confidence intervals were obtained with 100‐block bootstrap and SFS inference on these bootstrapped datasets with realSFS.

### 2.5 Scan for *F*_ST_ Outliers, Diversity and AFS

To determine which individual SNPs may display signatures of deviation from the expected frequencies given genome‐wide differentiation between urban and non‐urban anoles, we used an adapted version of the fastPCA algorithm modified for low‐depth samples (Galinsky et al. 2016). We also estimated Bhatia's *F*_ST_ (‐whichFst 1) (Bhatia et al. 2013), an estimator more robust to small/unequal sample sizes, and performed the Population Branch Statistics (PBS) test implemented in ANGSD/realSFS to retrieve genomic regions displaying unusually high differentiation between the urban populations and two other groups, including the 16 non‐urban individuals sequenced in this study and a set of seven anoles from the broad Gulf Atlantic genetic group identified in a previous work to which the Louisiana populations belong (Bourgeois et al. 2019). This latter set was chosen to cover the broader genetic diversity of the entire genetic cluster. We also estimated Tajima's *D* and nucleotide diversity *π* using ANGSD/realSFS, including monomorphic sites to avoid biasing calculations of *π* (Korunes and Samuk 2021). We used windows of 50 kb and 5 kb with sliding windows of 10 kb and 500 bp, respectively. For the X chromosome (scaffold LGb and X‐linked scaffolds listed in [Rovatsos et al. 2014]), we used a haploid model in ANGSD (‐isHap 1).

### 2.6 Demographic Inference and Simulations

To confirm the lack of strong or ancient differentiation between urban and non‐urban anoles, we estimated the joint 2D‐SFS using realSFS for all sites in the genome (including monomorphic ones) and used fastsimcoal2.8 to fit a simple model of isolation with migration (IM). Similarly to mutation load analyses, we projected the urban population to a sample of 64 haplotypes and the non‐urban population to a sample of 24. We incorporated inbreeding in the model by averaging the *F* coefficients estimated by ngsF for each population. Parameters with the highest likelihood were obtained after 20 cycles of the algorithm, starting with 50,000 coalescent simulations per cycle, and ending with 100,000 simulations. This procedure was replicated 50 times, and the set of parameters with the highest final likelihood was retained as the best point estimate.

We used the inferred demographic model to run neutral and non‐neutral coalescent simulations with ms (Hudson 2002) and msms (Ewing and Hermisson 2010). For positive selection, we used a very strong selection coefficient, guaranteeing fixation (advantageous *s* = 1, corresponding to a fitness increase of 100% in the urban population). We approximate strong background selection and inbreeding by scaling down effective population sizes in the neutral simulations by a factor of 10 (see, for example, Christe et al. 2016; Fraïsse et al. 2021), while keeping all other parameters the same. We estimated Bhatia's *F*_ST_ from the ms output using the sci‐kit allel package (v1.3.3) in Python 3.8.

### 2.7 Imputation of Missing Genotypes

The methods we used to examine genome–environment associations and spatial structure require complete genotype data. To address missing genotypes, we used STITCH v1.7.3 (Davies et al. 2016), a reference panel‐free imputation method that leverages haplotype structure and genomic alignments to infer genotypes from low‐depth sequencing data.

We first identified positions for imputation using a VCF file generated with bcftools, filtering out SNPs with more than 40% missing data and a site Phred score below 20. To assess imputation accuracy, we downsampled a non‐urban individual (AC8‐8) from 13× to 2× coverage, matching the depth of most individuals sampled in the New Orleans area. We then compared imputed genotypes to original high‐coverage genotypes using a confusion matrix and estimated the correlation coefficient (r^2^) between imputed and true genotypes.

STITCH accuracy is mainly governed by two parameters: *K*, the number of founder haplotypes, and *S*, the number of replicates used to average inference. We set the nGen parameter to 4 × *N*_e_/*K* (with *N*_e_ = 25,000) to approximate the recombination rate between ancestral haplotypes. STITCH is expected to be robust to moderate misspecification of this parameter. We tested combinations of *K* (4–24) and *S* (1–10) and retained the configuration that optimised both runtime and imputation accuracy (S = 10, K = 8). Imputation showed good performance, with a correlation coefficient of 0.84 between actual and imputed genotypes, as illustrated by the confusion matrix in Figure S1.

### 2.8 Spatial Barriers to Gene Flow Between Urban and Non‐Urban Anoles

To test for spatial barriers to gene flow between urban and non‐urban anoles, we used FEEMS v1.0.0 (Marcus et al. 2021) a fast method for detecting deviations from a strict isolation‐by‐distance model. We filtered out imputed SNPs with a minor allele frequency (MAF) < 0.05 and thinned the dataset to one marker every 50 kb to reduce linkage disequilibrium.

To select the optimal value of the regularisation parameter (*λ*), we followed the recommended leave‐one‐out cross‐validation procedure, testing 100 *λ* values on a logarithmic scale from 1.0 × 10^−4^ to 1000. We retained the *λ* value with the lowest cross‐validation error (*λ* = 38.53) for construction of the resistance map.

### 2.9 Genome‐Environment Association Scans

We used LFMM2 (Caye et al. 2019) to investigate associations between imputed genotypes and a range of environmental and urban features. LFMM2 accounts for population structure and unobserved confounding effects by incorporating latent factors into its association models.

To quantify environmental variation across sites, we compiled 22 landscape variables characterising both climate and urbanisation. These variables were also used in Winchell's study (Winchell et al. 2023). Specifically, we included the full set of 19 BIOCLIM climatic layers (v2.1 [Fick and Hijmans 2017]) at 30 arc‐second (~1 km^2^) resolution and radiance data from the NOAA Global Nighttime Lights dataset (F16_20100111‐20110731_rad_v4 GeoTIFF; https://www.ncei.noaa.gov/products/dmsp‐operational‐linescan‐system). Additionally, we incorporated two high‐resolution (30 m^2^) land‐cover layers: impervious surface cover (Homer et al. 2004) and canopy cover (Coulston et al. 2012), both from 2015. All variables were averaged within a 1 km buffer around each sampling point.

We reduced the dimensionality of the 19 BIOCLIM variables (bio1 to bio19) via PCA and retained the first three axes, which together explained 78.9% of the total variance (Table S2) (50.6%, 37% and 7.8% for PC1, PC2 and PC3, respectively). PC1 was mostly correlated positively with bio2 (diurnal temperature range), bio7 (annual temperature range), and bio19 (precipitation of the coldest quarter). PC2 was mostly correlated negatively with bio12 (annual precipitation) and bio14 (precipitation of the driest month), while being positively correlated with bio5 (Max Temperature of Warmest Month) and bio10 (Mean Temperature of Warmest Quarter). PC3 was mostly correlated negatively with bio8 (Mean Temperature of Wettest Quarter) and bio17 (Precipitation of Driest Quarter).

A separate PCA on the three urban‐related variables yielded a single retained axis (urban PC1, 88.6% of variance), capturing the dominant urbanisation gradient. This axis was positively correlated with canopy cover and negatively correlated with impervious surface cover and nightlight.

LFMM2 was run using three environmental PCs, one urban PC and three individual urban‐related landscape indices. We also performed a multinomial analysis using the three bioclimatic PCs and the urban PC as predictors. We used three latent factors in LFMM2 to correct for population structure, on the basis of a PCA of all 103 imputed Louisiana individuals. The number of factors was chosen on the basis of a visible drop in the percentage of explained variance between PC3 and PC4.

LFMM2 outputs inflation‐corrected *p*‐values. After confirming their approximate uniformity under the null, we applied a Benjamini–Hochberg false discovery rate correction and retained SNPs with adjusted *p*‐values ≤ 0.01 as outliers.

### 2.10 Outlier Properties

On the basis of the result of the simulations, we extracted candidate loci for selection in the urban population on the basis of their PBS and *F*_ST_ values. We used BEDTOOLS v2.30.0 to extract overlapping genes from the green anole annotation. We then used the R package GOfuncR (Grote 2017) to perform Gene Ontology (GO) enrichment analyses while correcting for gene length, since long genes overlapping many markers may be more likely to be identified by a genome scan.

We used the R package IWTomics (Cremona et al. 2018) to compare the properties of candidate windows for selection with the rest of the genome. The method uses Functional Data Analysis to identify positions along curves of measurements (e.g., time series or genomic positions) that display significant deviations between groups. For PBS/*F*_ST_ outliers, we centred the candidate regions on the peak value, and extracted 100 kb flanks (for a total of 100 + 50 + 100 kb). For candidate genes, we also extracted 250 kb regions covering the genes of interest. All other 250 kb regions were used as background. We excluded overlapping intervals. We used a 5 kb resolution for each 250 kb region, splitting them into 50 windows (5 kb × 50).

We obtained lists of candidate genes for selection from previous studies (Winchell et al. 2023) or by using AmiGO to extract and manually curate GO terms of interest, using the keywords “response to heat”, “locomotion”, “behaviour” and “immunity” (Table S3). We used GOfuncR to retrieve the associated genes. To test whether these candidate genes displayed significant differences in summary statistics, we used the package regioneR (Gel et al. 2015) to perform blockwise‐permutations tests on 50 kb sliding windows overlapping the genes of interest. We used the circularRandomizeRegions function that takes a set of genomic intervals, circularises each chromosome and rotates intervals along chromosomes randomly. This approach, therefore, maintains clustering and gene length in the pseudo‐observed data set. We used 1000 permutated data sets and used the proportion of permutations above or below the observed value to estimate a *p*‐value.

## 3 Results

### 3.1 Relatedness, Inbreeding and Structure

**FIGURE 1:**
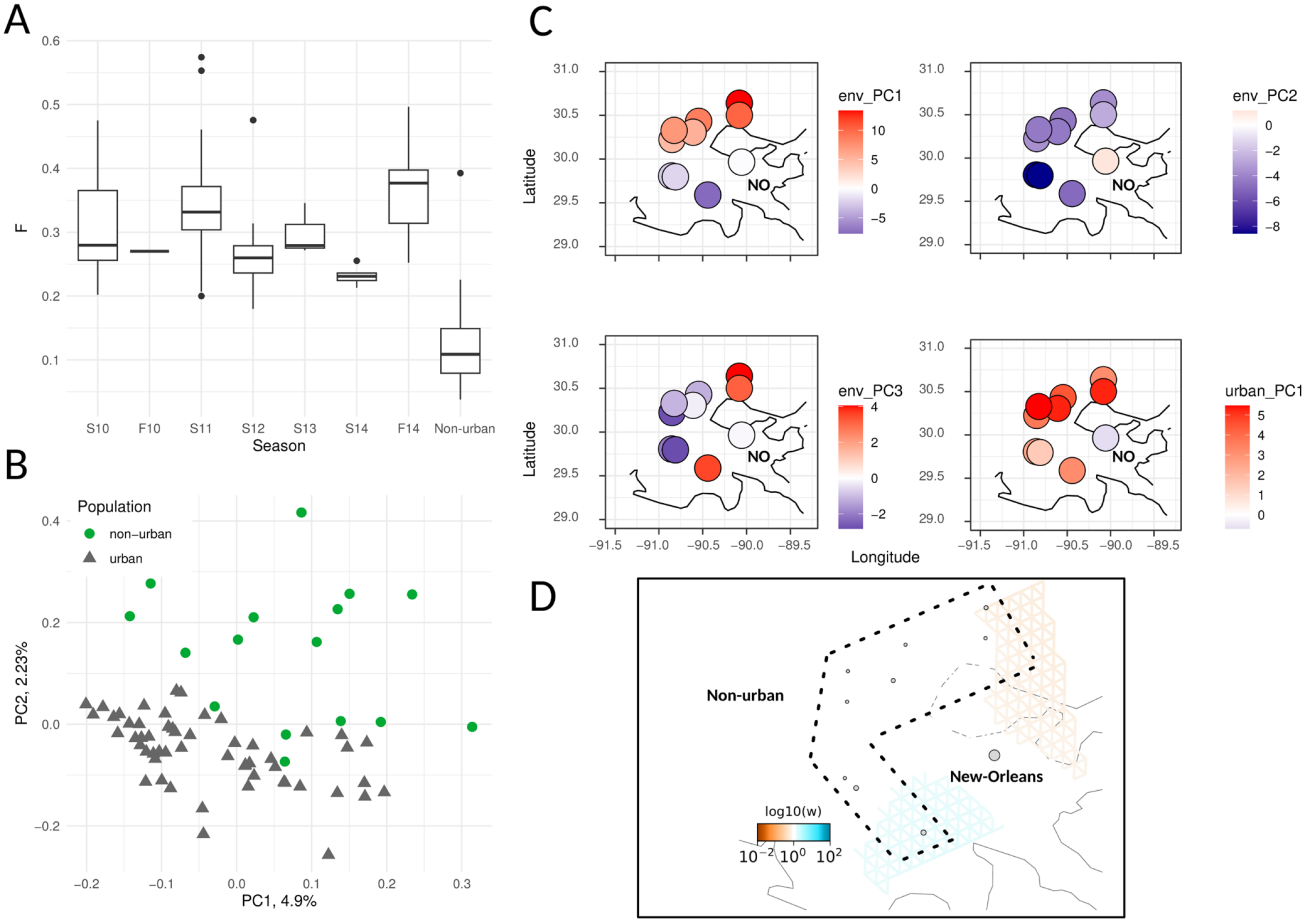
(A) Individual inbreeding coefficients (*F*) estimated by ngsF and plotted over sampling seasons for urban anoles. Non‐urban anoles are grouped under the boxplot titled “Non‐urban”. (B) Principal component analysis (PCA) on genomic data showing weak structure for urban and non‐urban anoles. (C) Projections of PC scores for PCA summarising bioclimatic data (env_PC) and the three urban features (urban_PC1) collected for Louisiana samples. New Orleans is indicated by “NO”. env_PC1, env_PC2 and env_PC3 explain 50.6%, 37% and 7.8% of the total variance, respectivelyurban_PC1 explains 88.6% of variance, showing a positive correlation with canopy cover and negative correlations with impervious surface cover and nightlight (see Section 2). (D) Effective migration surfaces obtained with LFMM2. Blue and orange areas correspond to increased and decreased migration compared to the expectation under a strict isolation‐by‐distance model, respectively.

A test of individual inbreeding (*F*) suited for low‐depth data showed high values in urban anoles found in Washington Square Park compared to non‐urban ones (Figure 1A), with a small but significant effect of sampling season in urban anoles (two‐way ANOVA on Box‐Cox transformed data, *λ* = 1.42, *F*_urban_ = 116.5, *F*_season_ = 2.7, *p* < 2.2 × 10^−16^ and *p* = 0.02 respectively). Nucleotide diversity was lower in urban anoles than in non‐urban ones (non‐overlapping 50 kb windows, average *π* = 3.9 × 10^−4^ and 5.5 × 10^−4^ respectively, Mann–Whitney paired test, *p* < 0.001). Divergence between the two groups was low (Bhatia's average *F*_ST_ = 0.027, median = 0.017, s.d. = 0.033, 95th percentile: 0.093). Low differentiation with the countryside was further highlighted by a principal component analysis on urban and non‐urban samples (Figure 1B). For urban anoles, we could identify 53 pairs of individuals with Θ > 0.0884, the lower confidence interval bound of this statistic for first and second degree relatives. The KING‐robust estimate was clearly biased upwards because of inbreeding (Figure S2) but also identified these pairs as related. We did not detect close relatives among non‐urban anoles using Θ > 0.0884.

Anoles from New Orleans were clearly distinct from neighbouring localities in both bioclimatic and urban‐related features (Figures 1C and S3). Urban individuals stood out as environmental outliers, occupying areas with a higher percentage of impervious surface and reduced canopy cover. They also exhibited higher scores on the second environmental principal component (see Section 2), which captured variation related to lower precipitation (with BIO12, BIO14, and BIO17 nearly collinear with this axis) and higher temperatures during the warmest months (primarily BIO5 and BIO10).

**FIGURE 2:**
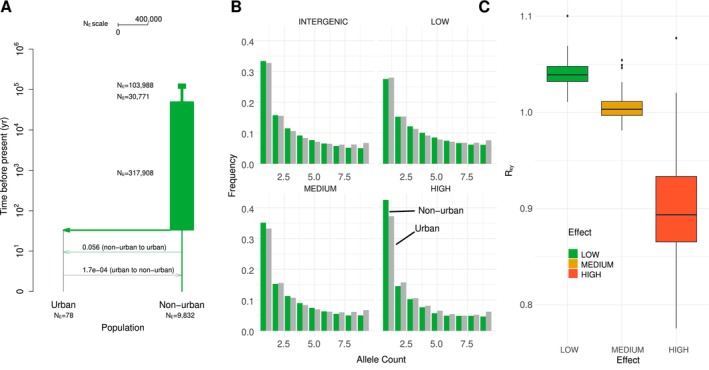
(A) Maximum Likelihood parameter estimates for an Isolation‐with‐Migration model fitted by fastsimcoal2.8. Times and population sizes are shown on a log10 scale. (B) Allele frequency spectra for variants classified by SNPEff as intergenic, or with low, intermediate and high effects. To facilitate comparisons between the urban and non‐urban groups, both spectra were projected down to 10 alleles. (C) Estimates of mutation load ($R_{\mathrm{XY}}^{\prime }$) estimated for the urban anoles, normalised by the load in non‐urban anoles. Boxplots correspond to the distributions obtained for 100‐block bootstrap replicates for each category of markers.

Despite these environmental contrasts, we did not detect strong barriers to gene flow between urban and non‐urban populations. This was further supported by our FEEMS analysis (Figure 1D), which revealed no pronounced barriers or corridors to gene flow. However, Lake Pontchartrain coincided with a region of relatively lower effective migration. This lack of strong structure was further confirmed by demographic inference. The joint frequency spectrum of unrelated individuals (49 urban, 28 non‐urban) supports low differentiation (Figure 2A). An Isolation‐with‐Migration model implemented in fastsimcoal2 inferred high levels of gene flow and a 32‐year time since divergence, assuming a mutation rate of 1.7 × 10^−9^ substitutions/year and a generation time of 1 year (Tollis et al. 2018). Nevertheless, we advise caution in interpreting too strictly these estimates, given the difficulty in estimating demographic parameters for recently diverged populations with methods using the SFS.

### 3.2 Loss of Mutation Load for Variants of Large Effects in Urban Anoles

An examination of allele frequency spectra for four categories of loci shows a clear increase of singletons and rare variants for loci classified as having moderate and high effects compared to intergenic loci and loci of low effect (Figure 2B). Estimates of $R_{\mathrm{XY}}^{\prime }$ show a slight increase in mutation load for variants of low and moderate effect, but a decrease in mutation load for variants of high effect (Figure 2C), suggesting that some purging occurred because of higher inbreeding and exposure of deleterious variants at the homozygous state. The higher proportion of singletons in the non‐urban population nonetheless suggests that selection on variants that remained after purging is less efficient in the urban population.

### 3.3 Bottom‐Up Scans for Positive Selection: Differentiation Scans

**FIGURE 3:**
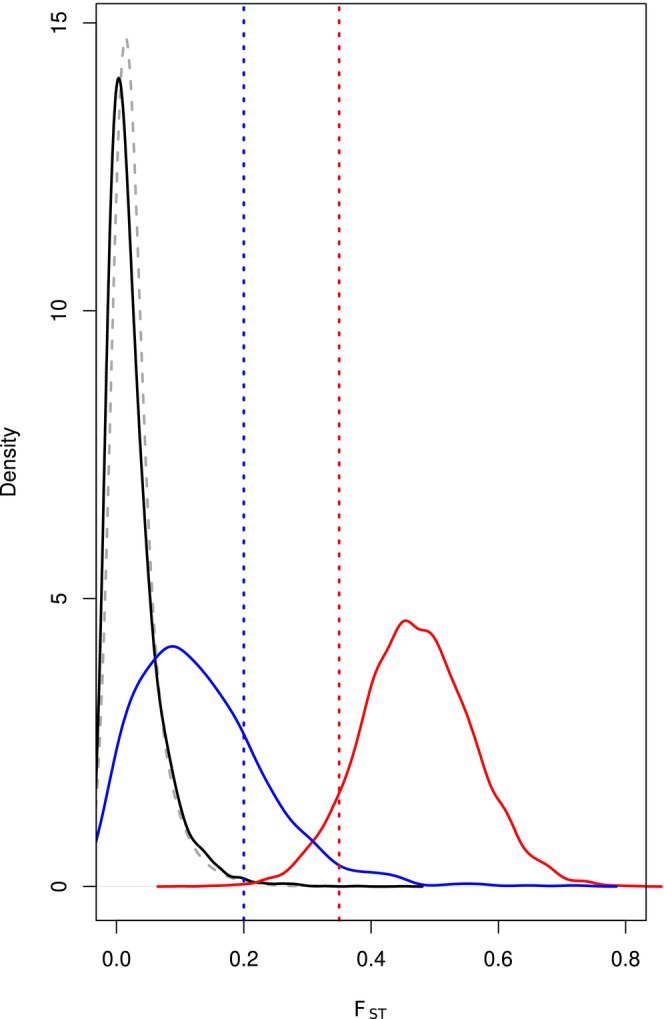
Distribution of *F*_ST_ between urban and non‐urban anoles for 50 kb windows simulated assuming neutrality (black line) or approximating strong background selection by scaling effective population sizes (*N*_E_background_ = 0.1 × *N*_E_neutral_, blue line). Simulations of a selective sweep that would guarantee fixation of a favourable allele in the urban population (*s* = 1) are shown in red. Genome‐wide estimates are given as a grey dotted line. Thresholds at 0.2 and 0.35 are indicated by dotted vertical lines.

To define thresholds for our tests of selection, we performed 10,000 simulations for three possible categories of 50 kb regions: neutral, under strong background selection and inbreeding and under strong positive selection in the urban population. Simulations suggest that windows of high *F*_ST_ between urban and non‐urban anoles may occur in the case of strong background selection and inbreeding, even in a context of low population structure (Figure 3).

On the basis of these simulations, we set two thresholds for *F*_ST_: one at 0.2, the other at 0.35. The first threshold corresponds to a value that discriminates between pure neutrality and strong reduction in diversity because of background selection and inbreeding, whereas the second was chosen to select candidates for strong positive selection while limiting the inclusion of windows under very strong purifying selection. We select windows as candidates if they also display a PBS value in the top 95th percentile, to avoid including signatures of selection in the non‐urban anoles.

**FIGURE 4:**
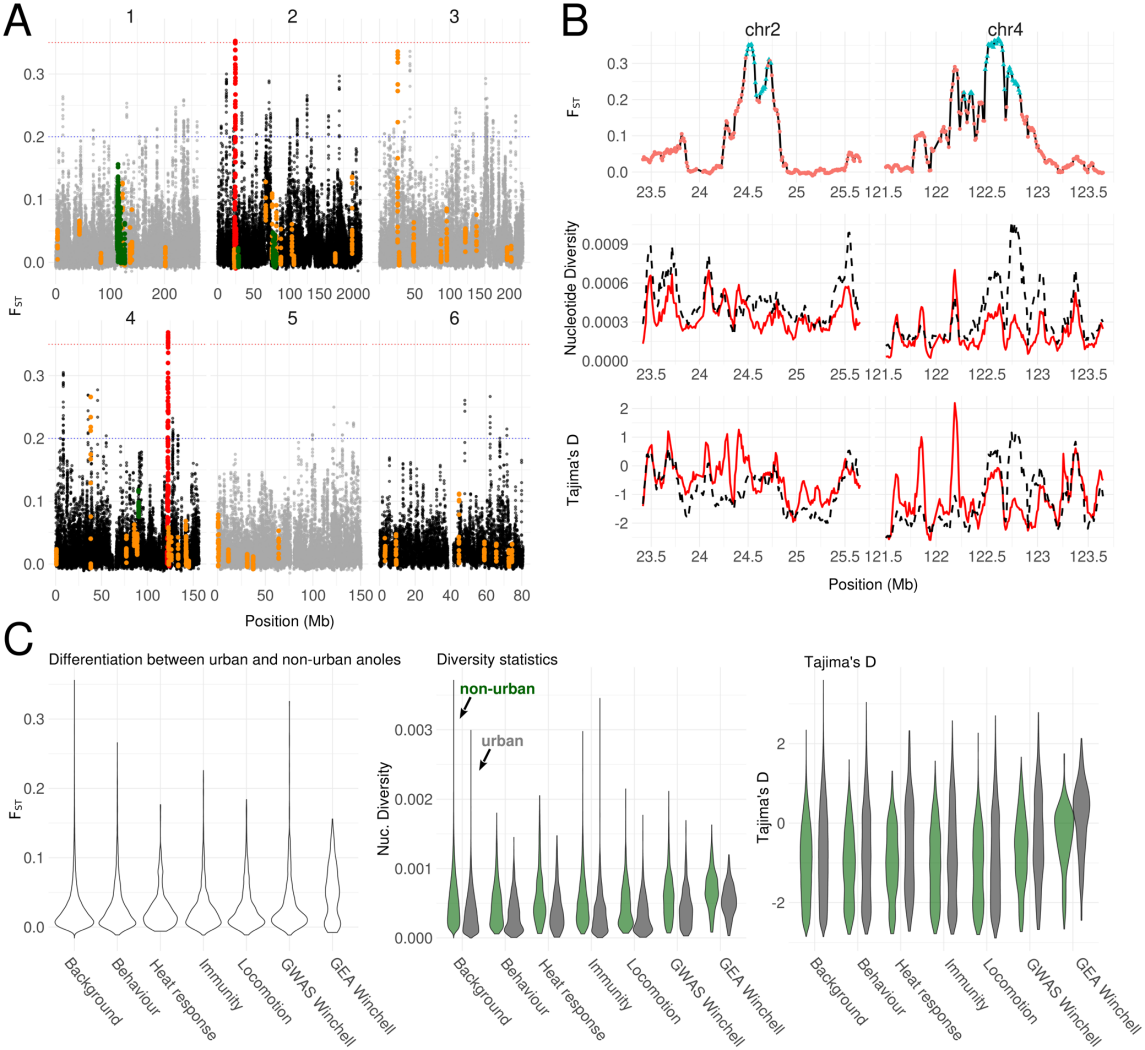
(A) Manhattan plot of genome‐wide *F*_ST_ between urban and non‐urban anoles from Louisiana, in 50 kb windows, sliding by 10 kb. Winchell's GEA candidate genes are highlighted in green and Winchell's GWAS genes in orange. The two regions above the *F*_ST_ threshold of 0.35 are shown in red. (B) Zoom on the two regions with scans for selection, displaying *F*_ST_, nucleotide diversity and Tajima's *D*. *F*_ST_ windows being also outliers for the PBS test in urban anoles are highlighted as blue triangles. Black dotted lines show values for the non‐urban population, red continuous lines correspond to the urban population. (C) Properties of 50 kb windows overlapping different categories of candidate genes (non‐urban on the left, urban on the right for each pair of violin plots).

Using an *F*_ST_ threshold of 0.2, we could identify 45 candidate regions for selection, covering 63 coding genes associated with a GO term (Figure 4A, Table S4). An examination of the coverage or recombination rate curves centred on these putative selective sweeps did not reveal significant differences when compared to background regions (functional data analysis, all *p* > 0.05). Candidate regions also did not differ in absolute divergence (*d*_*XY*_) between two divergent genetic clusters studied in a previous work (Bourgeois et al. 2019). We did not observe any clear drop in diversity nor Tajima's *D* in the urban population.

A GO enrichment analysis on these regions did not reveal any significant terms for biological processes after correction for multiple tests (Table S5). Before correction, many terms linked to cardiac activity could be retrieved with *p*‐values < 0.01, but correspond to only two genes on chromosomes 1 and 5, *KCNQ1* and *ANK2*. *KCNQ1* is a member of the potassium voltage‐gated channel subfamily Q member 1, which plays a function in social behaviour and corticosterone secretion among other terms; it is also involved in cardiac activity. *ANK2* stands for ankyrin 2, a gene that also plays a role in cardiac function.

Using an *F*_ST_ threshold of 0.35, we identify two divergence blocks, one on chromosome 2 (position 24,460,000 to 24,760,000), and the other on chromosome 4 (position 122,250,000 to 122,850,000). The block on chromosome 2 did not overlap any annotated coding gene, the nearest being *NR2F1* (85 kb in 3′) and *ARRDC3* (1.1 Mb in 5′). *NR2F1* belongs to the nuclear receptor subfamily 2 group F and plays a role in eye development. *ARRDC3* stands for arrestin domain containing 3 and plays a role in regulating cell‐surface expression of adrenergic receptors. The block on chromosome 4 overlapped with *RH2*, an opsin gene. In both cases, we could observe a drop in nucleotide diversity in the urban population compared to non‐urban values (Figure 4B).

### 3.4 Top‐Down Scans for Positive Selection: Differentiation Scans

We analysed diversity and divergence at candidate genes relevant to urban adaptation and tested for potential signals of parallel adaptation. To do this, we focused on candidate genes previously associated with urban adaptation in *Anolis cristatellus* (Winchell et al. 2023). These included two categories: (1) Winchell's GEA gene**s**, a set of 33 genes identified through Genome‐Environment Association (GEA) studies, and (2) Winchell's GWAS genes, a broader set of 93 genes associated with urban‐relevant phenotypes identified through genome‐wide association studies (GWAS). In addition, we examined gene sets linked to relevant biological processes on the basis of GO terms, including response to heat, locomotion, behaviour and immunity (see Section 2 and Table S3 for the full list of GO terms).

Only 1 annotated gene overlapped between Winchell's GWAS genes and the 45 candidate regions for selection using the *F*_ST_ threshold of 0.2. This gene, *PAX3*, is a 148 kb gene on chromosome 3 involved in the development of anatomical structures and the nervous system. Another Winchell's GWAS gene on chromosome 4, *NPC1*, showed an *F*_ST_ above 0.2, but a PBS score below the 95th genome‐wide quantile. This gene is a cholesterol transporter involved in cell autophagy, neurogenesis and behaviour. We also identify two other “behaviour” genes above both *F*_ST_ and PBS thresholds, *FBXL20* (chr6, behavioural fear response) and *KCNQ1* (chr1, social behaviour and corticosteron secretion among other terms, also involved in cardiac activity, see GO enrichment analysis above).

Winchell's GEA genes overlapped with windows showing significantly PBS and elevated *F*_ST_ only in pairwise comparisons involving the urban population, but not in the comparison between non‐urban anoles and the outgroup (Figure 4C, *F*_ST_ = 0.047 vs. 0.017; PBS = 0.039 vs. 0.02, blockwise‐permutation tests, *p*‐value < 0.001). We did not observe any significant difference in coverage of filtered sites across 50 kb windows (median coverage for GEA: 41,824 sites per window; background genes: 41,437/50,000, blockwise‐permutation test, *p*‐value = 0.4).

Finally, urban anoles showed reduced nucleotide diversity compared to non‐urban anoles, alongside an increase in Tajima's *D*. Within both urban and non‐urban populations, Winchell's GEA genes exhibited significantly higher Tajima's *D* and nucleotide diversity than background genes (blockwise‐permutation tests, all *p* < 0.001).

### 3.5 Bottom‐Up Scans for Positive Selection: Genome‐Environment Association

Selection acting on standing variation may leave narrow genomic signatures that are difficult to detect using window‐based tests. Although the New Orleans population clearly stands out on the basis of urban‐associated environmental metrics, other populations exhibit a more continuous gradient of urbanisation. To assess genotype‐environment associations (GEA), we used LFMM2 with three latent factors—corresponding to significant principal components identified via PCA—on imputed genotype data. We tested associations with urban‐related variables (canopy cover, impervious surface percentage, night‐time light intensity and the first PC summarising these variables) and separately with the first three Bioclim PCs. We also ran a multivariate test (referred to as the ‘global test’) incorporating both urban and Bioclim principal component axes (three from Bioclim and one from urban data; see Section 2).

For clarity, we refer to the individual tests on urban metrics as ‘urban test’ and to the joint PC‐based test as the ‘global test’. Results from the urban tests showed substantial overlap, with many genes identified across multiple urban features (Figure S4). In contrast, there was less overlap with genes identified through Bioclim PCs associations. Additionally, 50 kb windows overlapping outlier SNPs tended to have elevated *F*_ST_ and PBS values, suggesting localised differentiation (Figure S5). This pattern, however, was not exclusive to urban variables, underscoring the utility of association tests to directly link environmental variation with genetic differentiation.

Using a false discovery rate threshold of adjusted *p* < 0.01 (Benjamini–Hochberg correction), we identified 1384 genes overlapping outlier SNPs in at least one urban test, and 5630 genes from the global test. Among the 6740 urban outlier SNPs, 14 were non‐synonymous, spanning 14 unique genes (Table S6). Although no Gene Ontology terms remained significant after multiple testing correction (Tables S7 and S8), several terms related to locomotion and immune response were significant prior to correction in the global test (Table S7).

**FIGURE 5:**
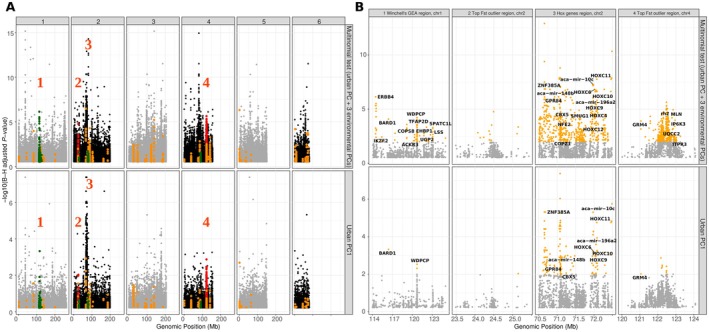
(A) Manhattan plots of Benjamini–Hochberg adjusted *p*‐values of association between environmental features and genotypes in 103 Louisiana anoles. Colour codes as in Figure 4. Numbers indicate regions displayed on panel B. (B) Zoom on four regions of interest, including the two *F*_ST_ outlier regions shown in Figure 4B. Top panels show results for the multinomial (‘global’) test, bottom panels show results for association with the first principal component of a PCA summarising three environmental variables (canopy cover, percentage of impervious surface and nighlight intensity).

Both the urban and global tests revealed a strong enrichment of outliers in a large genomic region on chromosome 2 (62–74 Mb) containing the *HOXC* gene cluster (Figures 5, S6 and S7). This region also showed increased differentiation. Regions with extreme *F*_ST_ also contained outliers—especially on chromosome 4—highlighting the consistency between outlier and GEA tests, and suggesting that GEA approaches can detect narrower signals of selection that may be missed by differentiation‐based scans alone.

### 3.6 Top‐Down Scans for Positive Selection: Genome‐Environment Association

Among the 1384 genes identified in the ‘strictly urban’ genotype‐environment association (GEA) tests, 74 candidate genes stood out on the basis of functional annotations (Table S9). Notably, four of these genes were also reported in Winchell et al., with two (*IKZF2* and *BARD1*) overlapping the same large region they identified in chromosome 1. This region also showed a high density of outliers in our global test (Figure 5B, Tables S10 and S11), supporting its potential role in broad environmental adaptation. The other two overlapping genes from Winchell's GEA analyses are listed in Table 1, alongside six additional genes found to overlap with outliers from their GWAS results.

**TABLE 1:** Summary of new candidate genes identified in this study.

Ensembl ID	Gene name	Chromosome	Start	End	Strand	Test	Description	Notes
ENSACAG00000028718	*KCNQ1*	1	68,988,624	69,393,295	−1	FST	Potassium voltage‐gated channel subfamily Q member 1	GWAS Winchell
ENSACAG00000013384	*ARRDC3*	2	23,370,803	23,389,669	−1	FST	Arrestin domain containing 3	
ENSACAG00000013324	*NR2F1*	2	24,822,744	24,838,409	1	FST	Nuclear receptor subfamily 2 group F member 1	
ENSACAG00000004981	*PAX3*	3	25,697,211	25,825,544	1	FST	Paired box 3	GWAS Winchell
ENSACAG00000000201	*NPC1*	4	38,209,186	38,261,159	1	FST	NPC intracellular cholesterol transporter 1	GWAS Winchell
ENSACAG00000016065	*RH2*	4	122,805,745	122,826,851	−1	FST	RH2 opsin	
ENSACAG00000010704	*ANK2*	5	142,556,975	142,772,454	−1	FST	Ankyrin 2	
ENSACAG00000017505	*FBXL20*	6	67,619,713	67,716,979	−1	FST	F‐box and leucine rich repeat protein 20	GWAS Winchell
ENSACAG00000010855	*HOXC10*	2	72,052,124	72,085,488	−1	GEA	Homeobox C10	In Locomotion gene list
ENSACAG00000007594	*ZNF385A*	2	70,694,205	70,881,570	1	GEA	Zinc finger protein 385A	In Locomotion and Behaviour gene lists
ENSACAG00000005073	NA	5	447,399	516,161	1	GEA		GWAS Winchell
ENSACAG00000017042	*KLHL23*	GL343362	544,682	549,131	1	GEA	Kelch like family member 23	GWAS Winchell
ENSACAG00000004981	*NA*	3	25,697,211	25,825,544	1	GEA		GWAS Winchell
ENSACAG00000013105	*CYP27B1*	2	66,873,290	66,891,936	−1	GEA	Cytochrome P450 family 27 subfamily B member 1	GWAS Winchell
ENSACAG00000001823	*NA*	4	85,612,001	85,635,151	1	GEA		GWAS Winchell
ENSACAG00000016163	*ITPR3*	4	123,042,437	123,184,648	−1	GEA	Inositol 1,4,5‐trisphosphate receptor type 3	GWAS Winchell
ENSACAG00000025864	*IKZF2*	1	115,423,904	115,538,818	−1	GEA	IKAROS family zinc finger 2	GEA Winchell
ENSACAG00000015438	*BARD1*	1	116,420,144	116,492,139	−1	GEA	BRCA1 associated RING domain 1	GEA Winchell
ENSACAG00000015126		2	81,152,464	81,247,292	−1	GEA		GEA Winchell
ENSACAG00000009873	*IDH1*	4	90,486,024	90,513,339	1	GEA	Isocitrate dehydrogenase (NADP(+)) 1	GEA Winchell

*Note:* GEA: Found as an outlier in urban tests. The Notes section specifies whether a gene was part of one of the gene lists established for top‐down analyses.

The large region on chromosome 2, which harbours strong signals of association, includes zinc finger protein 385A, a gene annotated for functions related to locomotion and behaviour. This gene displays a pronounced peak of association and is notable for being the only urban‐associated gene in our SNPEff analysis predicted to carry a loss‐of‐function mutation—specifically, an absent start codon.

Although genome‐wide tests revealed only limited evidence for functional enrichment of outlier‐associated genes compared to random permutations, we did observe a significant enrichment for genes annotated with Gene Ontology (GO) terms related to locomotion, particularly in association with nightlight (permutation test, *p* = 0.049), first Bioclim PC (*p* = 0.006) and the urban PC (*p* = 0.047). The multivariate (global) test showed marginal enrichment for outliers in Winchell's GWAS genes as well (*p* = 0.059).

## 4 Discussion

This study investigates the evolutionary effects of urbanisation on a population of *Anolis carolinensis* . This urban population is characterised by increased inbreeding and the associated purging of deleterious mutations through systematic inbreeding. Comparative genomic analyses revealed substantial divergence between urban and non‐urban populations, with patterns suggesting polygenic selection acting on traits related to behaviour, development and immune function. These findings highlight the role of urban environments in driving parallel adaptation across populations and underscore the need for more comprehensive sampling and modelling to elucidate the genetic basis of urban adaptation.

### 4.1 Habitat Fragmentation Increases Inbreeding and Purges Some Mutation Load

We observed clearly higher levels of inbreeding in urban anoles than in non‐urban individuals, which is likely the result of the relative isolation of the urban population, as well as its small effective population size (*N*_E_ = 78 individuals). This result is in line with a previous study using microsatellites on the same urban population examined here (Weber et al. 2021).

Populations evolving in urban habitats are expected to be fragmented and isolated from non‐urban ones. Moreover, small population sizes may lead to a reduced efficiency of purifying selection against deleterious mutations, increasing the load. However, we do not detect any strong barrier to gene flow between urban and non‐urban anoles, as highlighted by demographic inference, summary statistics, and FEEMS analysis. Our results rather align with the expectation that inbreeding increases the exposure of recessive deleterious mutations and purges the potential mutation load, particularly variants of strong effect. Both previous microsatellite studies and our results suggest so‐called ‘systematic inbreeding’, with *F*_IS_ > 0, a scenario that may be more efficient at purging deleterious alleles (Glémin 2003). Although some recent studies have been conducted on the mutation load of small populations in a conservation context (Dussex et al. 2021; Grossen et al. 2020; Hasselgren et al. 2024; Mathur and DeWoody 2021; Xue et al. 2015), this is, to our knowledge, the first evidence of purging in an urban context (Charmantier et al. 2024). More studies in urban contexts are needed to determine how general the pattern we observe may be.

The urban population of *A. carolinensis* studied here is under the effects of both genetic drift and purging, which results in a genome‐wide loss of heterozygosity, including potentially adaptive variation. However, we detected signals that are consistent with selection on standing variation in urban habitat (see below). Thus, it does not seem that the combination of purging and drift constrains selection to act upon a limited number of initially rare variants, at least in this context. It is, however, possible that these effects are mitigated by recurring gene flow to the urban population. Such ‘urban facilitation’ may be more frequent than previously expected (Miles et al. 2019) and may be important in restoring diversity. Another intriguing possibility is the expectation of increased mutation rates in urban populations (Johnson et al. 2024), which may contribute to refuelling diversity upon which positive selection may act.

### 4.2 Evidence for Convergent Evolution at the Gene Level in Urban Anoles

Our study highlights significant genomic divergence between urban and non‐urban populations of *Anolis carolinensis* . Regions of the genome showing differentiation between these populations also exhibit higher genetic diversity and elevated Tajima's *D*, compared to the rest of the genome. However, the urban population generally shows lower diversity and Tajima's *D* values than the non‐urban population. This pattern suggests that selection may have acted on preexisting genetic variation, indicating a soft sweep (Messer and Petrov 2013; Pennings and Hermisson 2006), or that overall diversity and Tajima's *D* are too low to provide a strong contrast with hard selective sweeps. Additionally, the higher diversity observed suggests that fixation of extended haplotypes in urban populations is rare, pointing to polygenic selection and phenotypic plasticity as the main mechanisms of adaptation, rather than simple, large‐scale changes in allele frequency at a few loci with major effects. This is further supported by the relative rarity of non‐synonymous variants in our GEA study, suggesting that more subtle changes in gene expression and regulation, rather than coding variation, drive adaptation.

By investigating candidate genes associated with adaptation to urban environments in another Anolis species, we observed increased genetic differentiation in the urban population of *A. carolinensis* . Although none of these genes surpassed the conservative *F*_ST_ threshold of 0.2, they showed higher divergence than expected by chance. These genes are enriched for functions related to immune response and inflammation, which aligns with previous findings of heightened injuries and stress in urban anoles (Tyler et al. 2016). Among these genes, two displayed signals of association in urban tests (*IKZF2* and *BARD1*). The entire region showed signals of association in our global test, indicating a broader adaptive response beyond just urban‐related pressures. Furthermore, we identified genes involved in behavioural regulation (such as *FBXL20* [Yao et al. 2011]) and developmental processes (such as *PAX3*), supporting the notion that urban adaptation involves changes in morphology, behaviour (Sol et al. 2020) and locomotion (Winchell et al. 2020). One particularly intriguing finding was the high *F*_ST_ observed near the *RH2* opsin gene, which could be involved in light detection and behavioural responses to the environment (Kelley and Davies 2016). Of particular interest was the high density of SNPs associated with urban and environmental features in a large region of chromosome 2, encompassing several *HOXC* genes. Although this region showed elevated *F*_ST_ in 50 kb windows, few surpassed our outlier thresholds. In contrast, a 5 kb window size aligned more closely with the GEA results (Figure S7), further highlighting the value of GEA for detecting subtler signals of selection. These findings call into question the assumption that urban adaptation is predominantly driven by strong, hard selective sweeps that drastically reduce genetic diversity.

The *HOXC* gene cluster is particularly interesting, given its importance in limb development and locomotion, traits that are under strong diversifying selection in anoles (Lailvaux 2020; Prado‐Irwin et al. 2019; Wade 2012; Winchell et al. 2016). For instance, *HOXC10* knockout mice have malformed pelvis and hindlimb bones—especially aberrant femur shape—and they display changed locomotor behaviour (Hostikka et al. 2009). These mice also lose lumbar motor neurons, linking *HOXC10* to both skeletal patterning and the motor circuitry that controls hindlimb movement. Similarly, *HOXC8* is crucial in limb development and motor neuron terminal differentiation (Catela et al. 2022). In both cases, limb morphologies shaped by *HOXC* genes (bone length, joint formation and ligament pattern) correlate with deficits in limb use and walking. To our knowledge, this is the first study highlighting *HOXC* genes as candidates for selection in anoles, although evidence for positive selection at other *HOX* clusters was found in a macroevolutionary study (Tollis et al. 2018).

Our findings add to the growing evidence of polygenic parallelism in urban environments, particularly when considered alongside the work of Winchell et al., who identified parallel genomic signatures in urban *Anolis cristatellus* . The overlap of candidate genes associated with urban adaptation—such as *PAX3*, *KCNQ1* and *FBXL20*—highlights shared functions in immunity, neural processes and development, consistent with the selective pressures imposed by urban habitats. The enrichment of such genes in urban anoles supports the hypothesis that similar selective forces, such as heightened injury risks, stress and novel behaviours, drive convergent evolution in *Anolis* species. However, it remains uncertain whether these signatures extend across species or ecomorphs. Among GEA outliers, only genes related to locomotion showed a significant enrichment, suggesting that selection in urban environments may act on a broader and more polygenic basis than what our tests are designed to detect. Rather than repeatedly targeting a small number of genes with moderate or large effects, our results support a model of genetic redundancy, where adaptation primarily draws on preexisting variation at many genes (Yeaman 2015). This interpretation is consistent with a scenario of functional, rather than genetic, convergence—as also proposed for other complex traits in natural populations of green anoles (Crawford et al. 2023). The limited number of genes for which convergence between *A. carolinensis* and *A. cristatellus* was found could also be related to the ancient divergence between these species (35–40 My ago) and to the fact that they are quite different in a number of ways that may affect their adaptation to urban habitats. These two species correspond to different ecomorphs (trunk‐ground for *A. cristatellus* , trunk‐crown for *A. carolinensis* ), which differ in morphological features, such as the length of their limbs, that could affect the way they occupy urban spaces. In addition, *A. cristatellus* is a tropical species, which is exposed to temperatures in Puerto Rico (particularly in urban habitats) that are much higher than the temperatures *A. carolinensis* experiences in continental USA.

Testing for genomic parallelism in more distantly related *Anolis* species or those with divergent ecologies could elucidate whether urban adaptation universally targets similar genetic pathways. Such studies would not only strengthen the case for convergent evolution but also clarify the links between genetic changes and the well‐documented phenotypic adaptations in urban anoles, offering a deeper understanding of the mechanisms underlying urban evolution.

### 4.3 Urban Habitat Adaptation: Potential Biases and Conclusions

After applying stringent filters on mapping quality and coverage depth, we found no systematic biases in genome coverage, suggesting that issues such as misalignment or paralogues are unlikely to explain the observed high *F*_ST_ values. However, factors like linkage disequilibrium and linked selection can reduce local genomic diversity, potentially leading to false positives in selection tests [(Hoban et al. 2016; Huber et al. 2016) but see (Matthey‐Doret and Whitlock 2019) on the relative insensitivity of *F*_ST_ to background selection]. Although we exercised caution in interpreting our results, the right‐shifted *F*_ST_ distribution in our simulations suggests that linked selection and inbreeding could bias tests for local adaptation. Nonetheless, on the basis of the properties of windows showing high *F*_ST_—such as the lack of differences in absolute divergence (*d*_*XY*_) or recombination rates between anole clades and even increased nucleotide diversity compared to background windows for Winchell GEA candidates—background selection and excess homozygosity because of inbreeding appear unlikely explanations.

One surprising observation was the absence of a significant drop in nucleotide diversity or Tajima's *D* in candidate regions. A combination of recurrent selection and migration could maintain and “recycle” haplotypes, resulting in high *F*_ST_, while preserving nucleotide diversity. The balance between selection and migration across environmental gradients has also been proposed as a mechanism by which regions under adaptive selection can display higher diversity (Jasper and Yeaman 2024). Such a process may extend beyond adaptation to urbanisation and involve recurrent selection on certain genes across various environments, promoting long‐term maintenance of genetic diversity. Although a hard sweep driven by new mutations in the urban population would typically reduce diversity and create clear genealogical signatures, such patterns are more elusive under soft sweeps and weak polygenic selection. If our findings reflect the recycling of preexisting variation, confirming the role of urban selective pressures may require denser spatial and temporal studies to track the establishment and evolution of anoles in urban environments.

Another key consideration is the scale of sampling, which plays a critical role in adaptation studies. In the absence of controlled conditions, attributing high *F*_ST_ specifically to urban adaptation remains challenging. Previous studies could have mistaken global signatures of selection for local phenotypic changes, thereby linking high differentiation with urban adaptation incorrectly (Bosse et al. 2019; Perrier and Charmantier 2019). To mitigate this, we compared our urban population with non‐urban individuals sampled from outside New Orleans and excluded regions showing differentiation across all comparisons, including those with out‐of‐Louisiana Gulf‐Atlantic individuals. We also ran SNP‐based scans of association with urban and environmental variables, increasing our sensitivity to narrow signals and specificity to urban‐related genes. Additionally, we examined candidate genes identified in other species, such as *A. cristatellus* . However, given the limitations in modelling, some of the regions we identified could still represent false positives or artefacts. Clear estimates of the selective advantage of specific urban alleles remain scarce (Charmantier et al. 2024), and future studies with denser sampling will be necessary to provide more robust estimates of selection coefficients for candidate genes in urban habitats.

In conclusion, our findings emphasise the important role urbanisation plays in shaping adaptive landscapes and suggest the potential for parallel evolution across urban populations. The strong differentiation and selection signals at loci related to behaviour and immune function indicate that these traits are key targets of selection in urban environments, supporting the idea that cities act as unique evolutionary hotspots. Further research should continue exploring the genetic basis of urban adaptation, particularly in relation to sexual selection and reproductive strategies in anole species.

## Author Contributions

S.B. funded and designed the study. S.L. designed sampling, collected samples and provided insights on the basis of his long‐term survey of *Anolis carolinensis* populations. Y.B. performed bioinformatic and population genetics analyses. Y.B. and S.B. wrote the first version of the manuscript, with input from S.L. All authors provided input at each step of the study and approved the final version of the submitted manuscript.

## Conflicts of Interest

The authors declare no conflicts of interest.

## Supporting information


Figure S1.–S7.

Table S1.–S11.

## Data Availability

Data accessibility: Raw sequence reads are deposited in the SRA (BioProject PRJNA1268161 and PRJNA533001 for anoles not in Louisiana). All the scripts used for this work are freely available as annotated scripts and Quarto notebooks at https://github.com/YannBourgeois/Scripts_urban_anolis_lowdepth. All analyses were carried out on the NYUAD Jubail HPC.
