# Supplementary material for: Urban Life Shapes Genetic Diversity in the Green Anole, Anolis carolinensis
Source: Mol Ecol. 2025 Jul 29;34(18):e70057. doi: 10.1111/mec.70057 (PMC12421488; doi:10.1111/mec.70057)
Supplement: Supplementary file 1 — Figure S1.–S7. [file MEC-34-e70057-s001.pdf]

Supplementary Figures

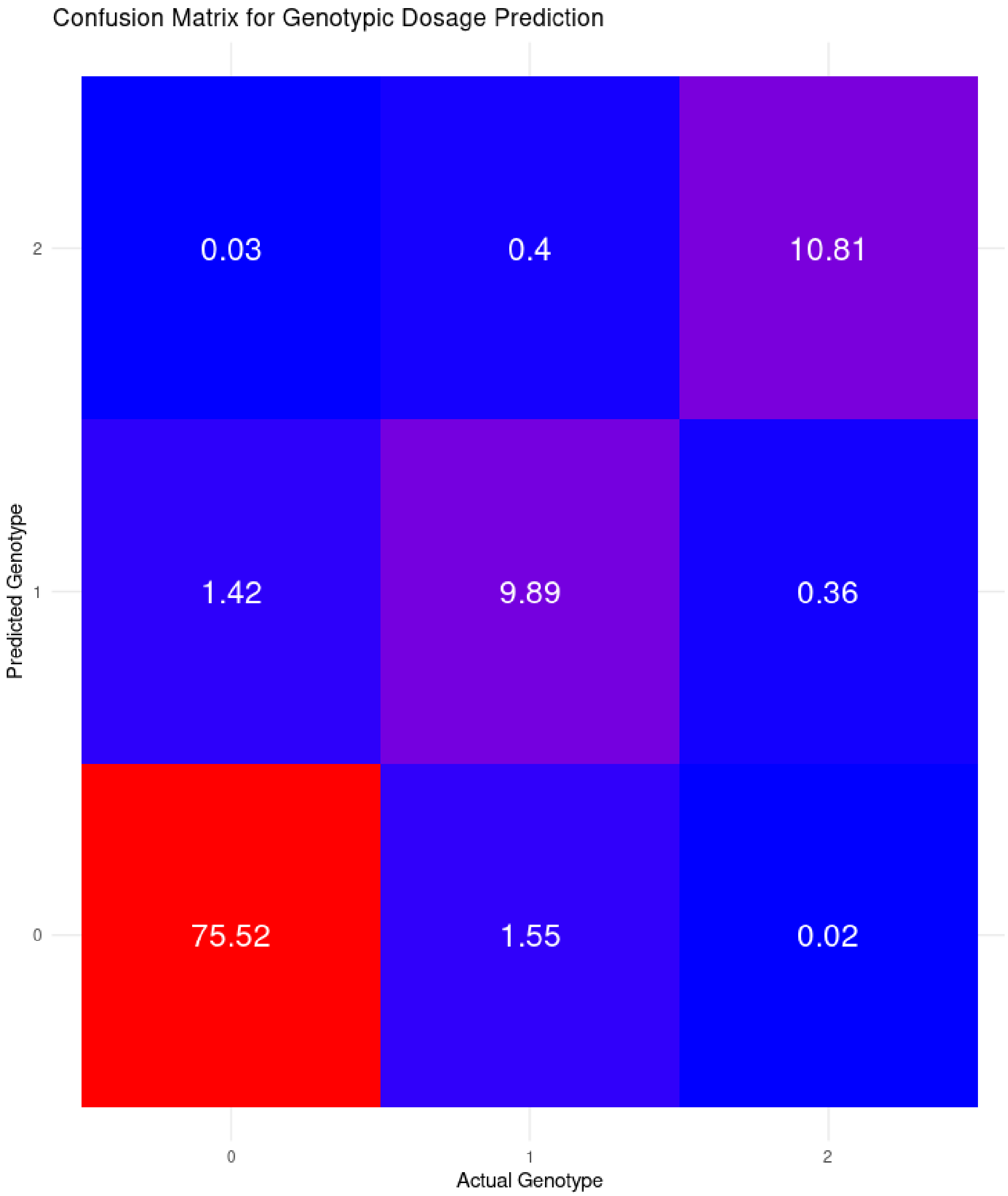

Sup. Figure 1: Confusion matrix for genotype imputation. The proportion of all genotypes falling in each category is indicated as a percentage. Actual genotypes are on the x axis, imputed genotypes in the y axis.

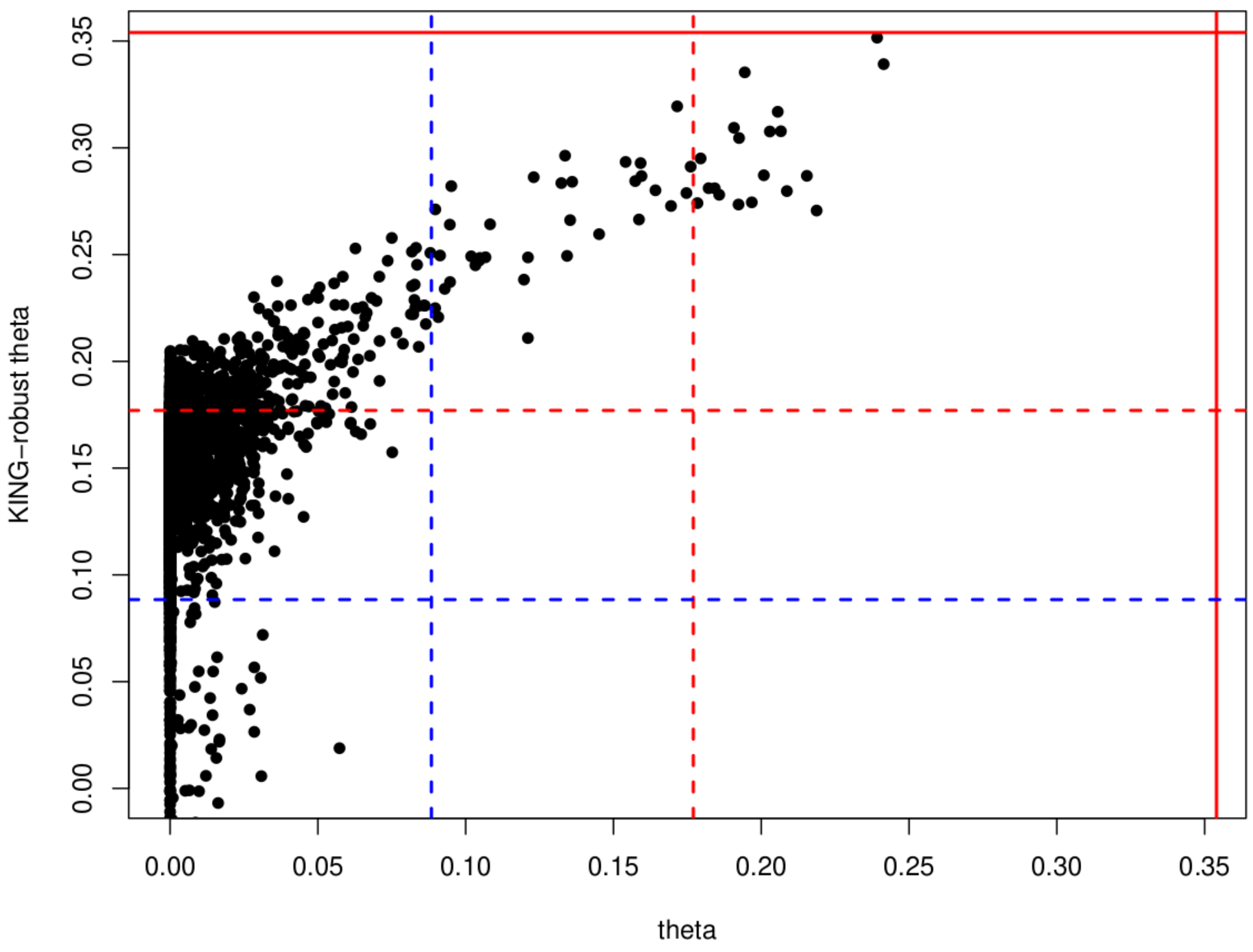

Sup. Figure 2. Estimators of relatedness. The thresholds indicate the confidence intervals for first (red) and second degree (blue) relatives. Note the inflation of KING-robust statistics.

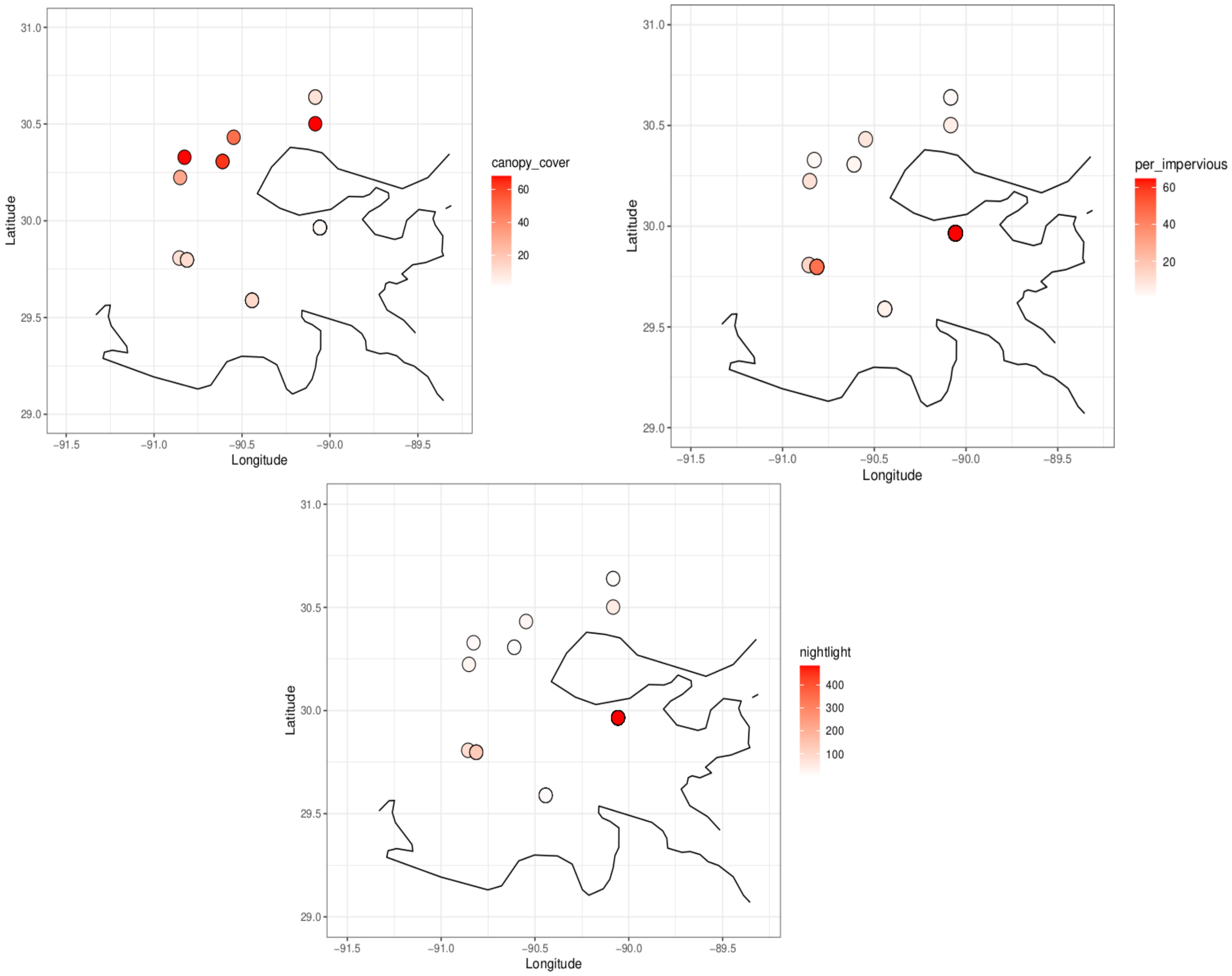

Sup. Figure 3. Spatial projection of urban statistics for each sampled locality.

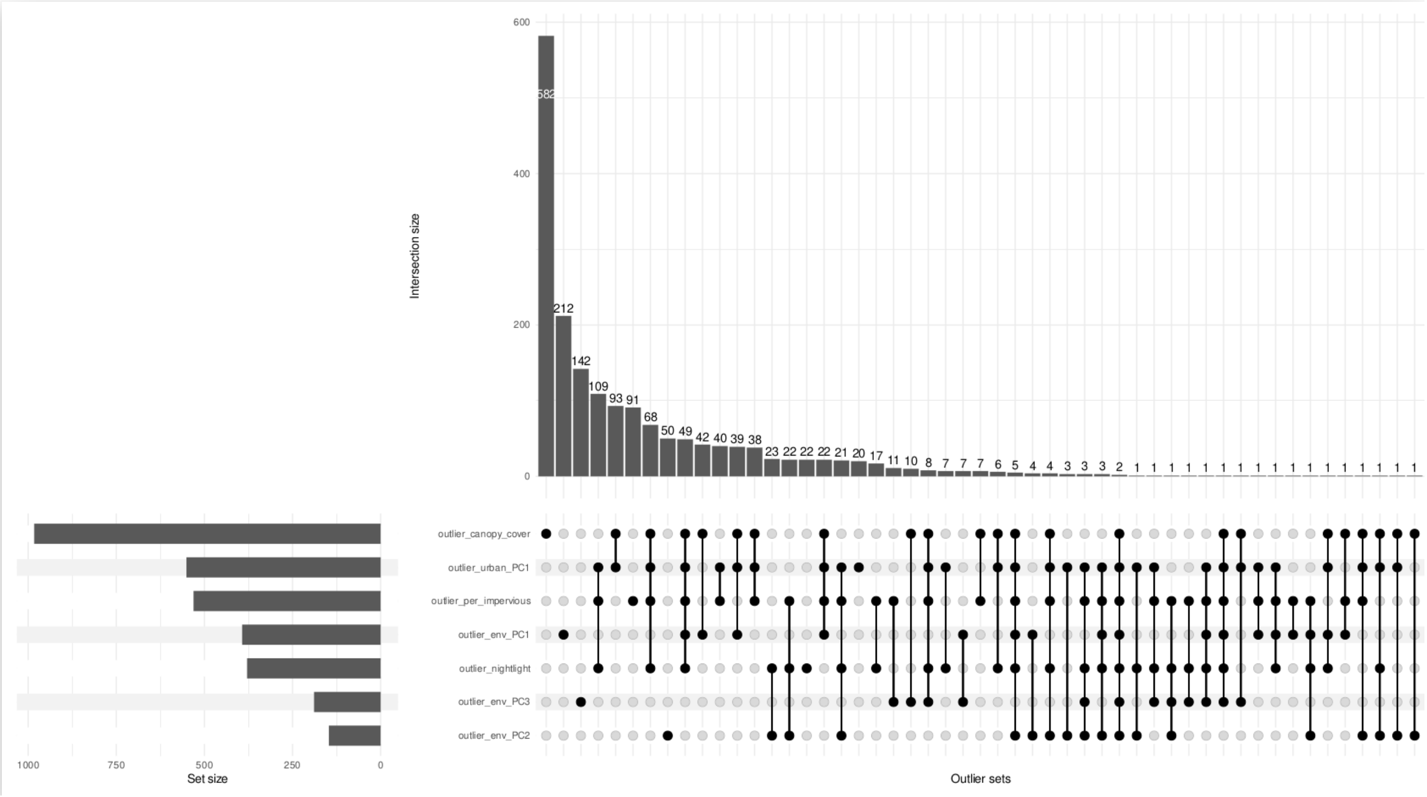

Sup. Figure 4. Overlap between univariate tests of selection in LFMM2 analyses.

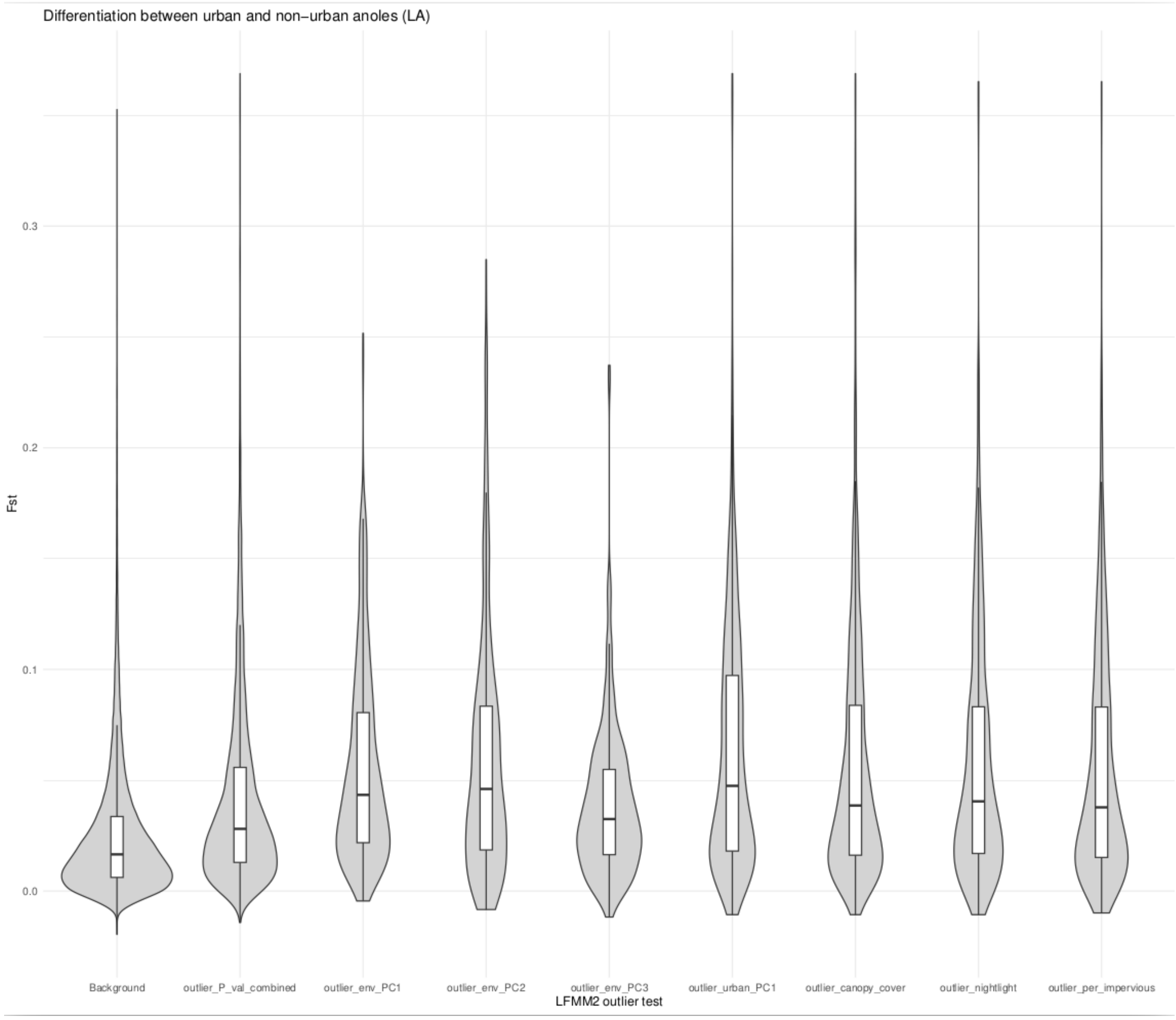

Sup. Figure 5. Violin plots of F_{ST} between urban and non-urban anoles for windows overlapping SNP outliers for each LFMM2 test.

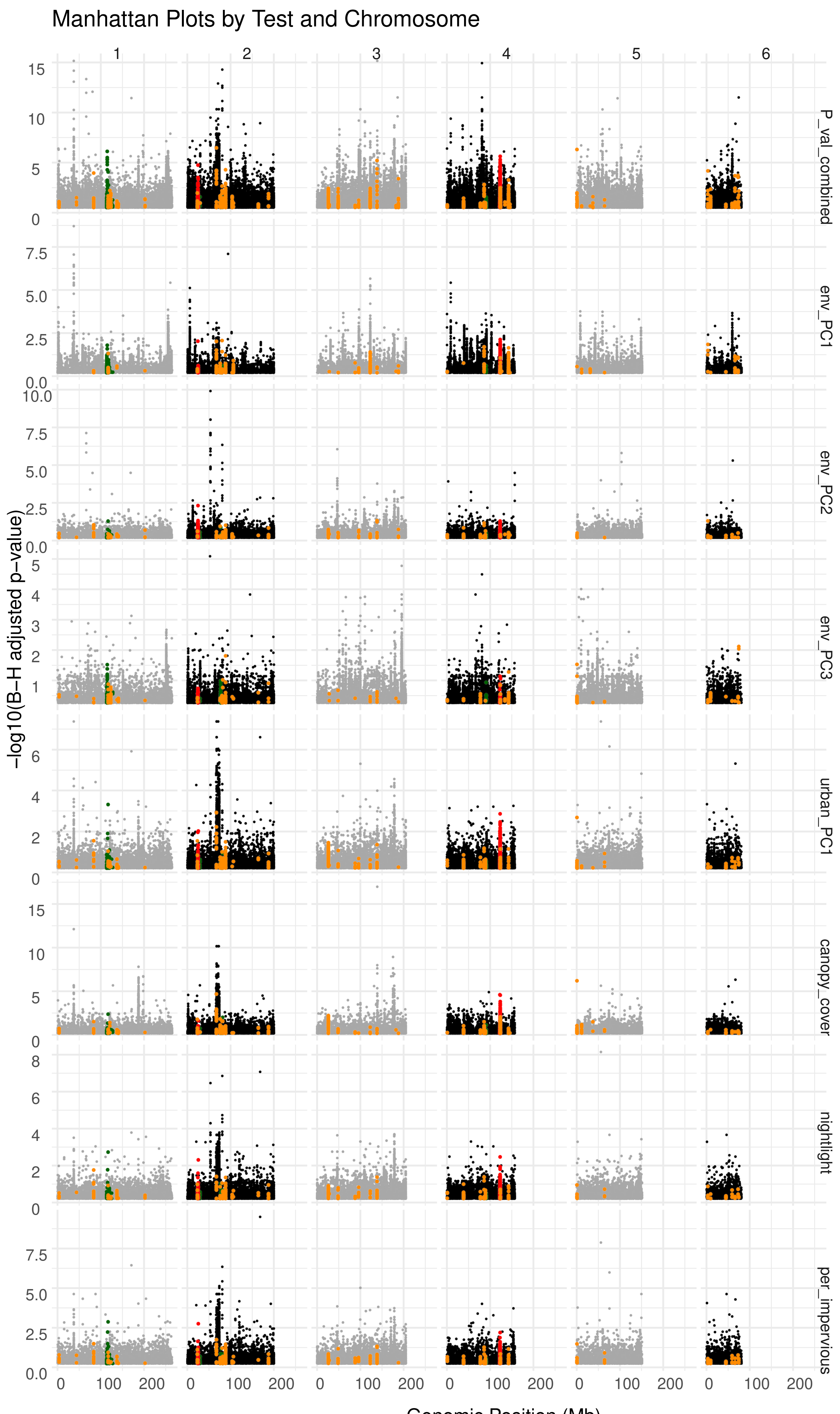

Manhattan Plots by Test and Chromosome
1
2
3
4
5
6
P_val_combined
env_PC1
env_PC2
env_PC3
urban_PC1
canopy_cover
nightlight
per_impervious
-log10(B-H adjusted p-value)
Genomic Position (Mb)

Sup. Figure 6: Manhattan plots of all LFMM2 association scans. Colour codes as in Figure 4. Blue dots indicate the position of behaviour-related genes.

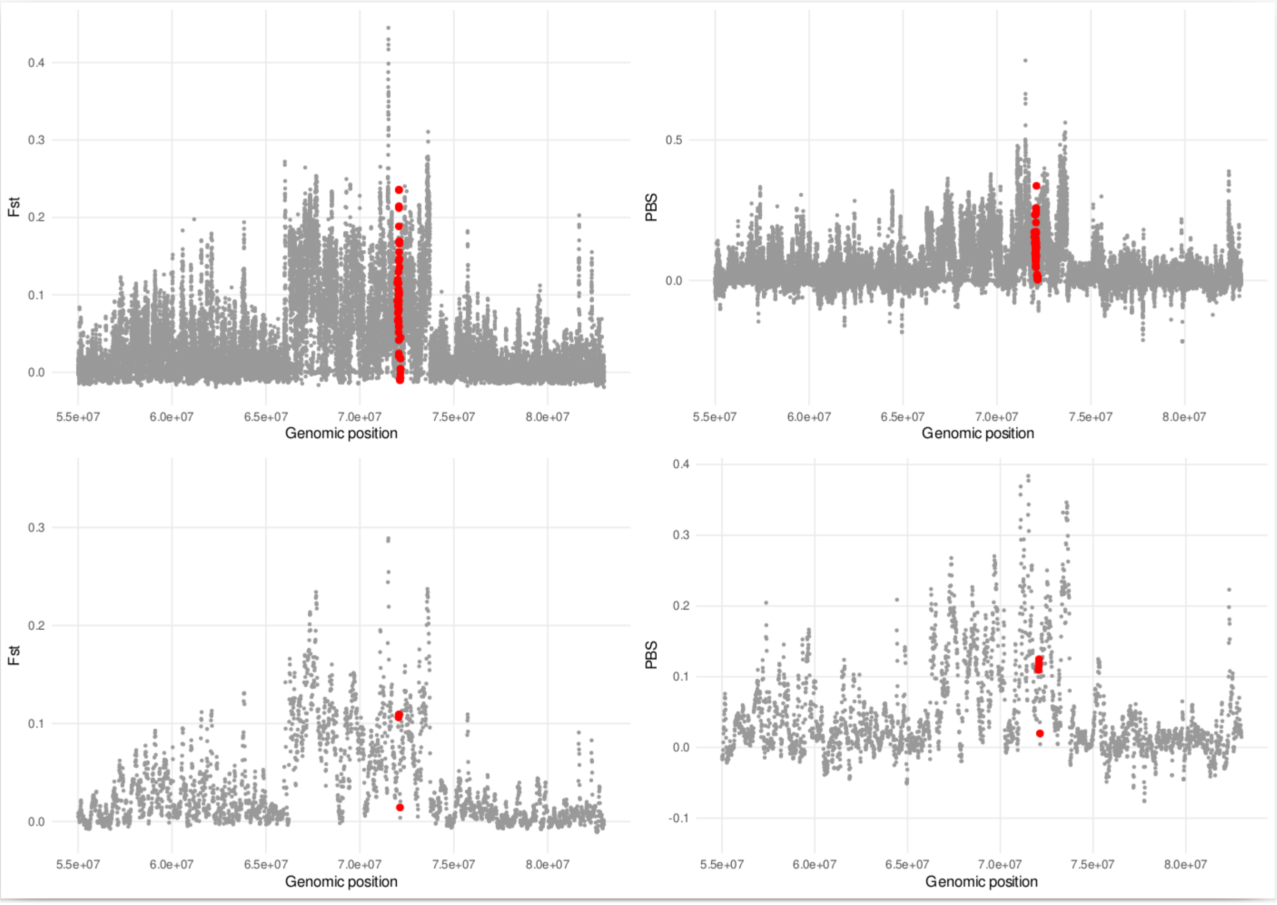

Sup. Figure 7: Detailed view of F_{ST} between urban and non-urban anoles, and PBS statistics in the large outlier region on chromosome 2. The red dots indicate the position of *HOXC* genes. Top panels: 5kb windows, overlapping by 1kb. Bottom panels: 50kb windows, overlapping by 10kb.
