# Supplementary material for: Urban Life Shapes Genetic Diversity in the Green Anole, Anolis carolinensis
Source: Mol Ecol. 2025 Jul 29;34(18):e70057. doi: 10.1111/mec.70057 (PMC12421488; doi:10.1111/mec.70057)
Supplement: Supplementary file 2 — Table S1.–S11. [file MEC-34-e70057-s002.zip › Sup_Tables_legends.docx]

Supplementary Table 1. List of samples included in this study. Geographic coordinates, average sequencing depth, coverage, and environmental data are provided for each individual.

Supplementary Table 2. Summary of the two Principal Component Analyses (PCA) performed on urban and bioclimatic variables. For each analysis, the loadings of each variable on the first three principal components are reported, along with the proportion of variance explained by each component.

Supplementary Table 3. List of Gene Ontology (GO) terms of interest and their classification into key biological functions: heat response, immunity, behavior, and locomotion.

Supplementary Table 4. List of genes overlapping with *F_ST_* outlier windows (threshold = 0.2).

Supplementary Table 5. Results of a Gene Ontology (GO) enrichment analysis for the genes listed in Supplementary Table 4. Reported terms correspond to biological processes (BP).

Supplementary Table 6. List of 14 non-synonymous SNPs significantly associated with urban variables in LFMM2 analyses.

Supplementary Table 7. Results of GO enrichment analysis for biological process (BP) terms among genes significantly associated in the global (multinomial) LFMM2 test.

Supplementary Table 8. Results of GO enrichment analysis for biological process (BP) terms among genes significantly associated in LFMM2 tests using individual urban variables.

Supplementary Table 9. List of 74 genes significantly associated in urban LFMM2 tests that also (i) have a GO term of interest from Supplementary Table 3, (ii) were identified in Winchell et al., or (iii) overlap with *F_ST_*  outlier regions using a threshold of 0.35.

Supplementary Table 10. List of 317 genes significantly associated in both urban and global LFMM2 tests and matching at least one of the following criteria: (i) associated with a GO term of interest from Supplementary Table 3, (ii) identified in Winchell et al., or (iii) overlapping with *F_ST_*  outlier regions (threshold = 0.35).

Supplementary Table 11. Comprehensive list of all outlier genes identified across all LFMM2 tests.
